# Team- and task-related knowledge in shared mental models in operating room teams: A survey study

**DOI:** 10.1016/j.heliyon.2023.e16990

**Published:** 2023-06-03

**Authors:** Tessa L. Verhoeff, Jeroen J.H.M. Janssen, Falco Hietbrink, Reinier G. Hoff

**Affiliations:** a Department of Anesthesiology, University Medical Center Utrecht, Heidelberglaan 100, 3584 GA, Utrecht, the Netherlands; b Department of Education, Utrecht University, Heidelberglaan 1, 3584 CS, Utrecht, the Netherlands; c Department of Surgery, University Medical Center Utrecht, Heidelberglaan 100, 3584 GA, Utrecht, the Netherlands

**Keywords:** Shared mental models, Team mental models, Teamwork, Operating room team

## Abstract

**Objective:**

The operating room is a highly complex environment, where patient care is delivered by interprofessional teams. Unfortunately, issues with communication and teamwork occur, potentially leading to patient harm. A shared mental model is one prerequisite to function effectively as a team, and consists of task- and team-related knowledge. We aimed to explore potential differences in task- and team-related knowledge between the different professions working in the operating room. The assessed team-related knowledge consisted of knowledge regarding other professions’ training and work activities, and of perceived traits of a high-performing and underperforming colleague. Task-related knowledge was assessed by mapping the perceived allocation of responsibilities for certain tasks, using a Likert-type scale.

**Design:**

A single sample cross-sectional study.

**Setting:**

The study was performed in three hospitals in the Netherlands, one academic center and two regional teaching hospitals.

**Participants:**

106 health care professionals participated, of four professions. Most respondents (77%) were certified professionals, the others were still in training.

**Results:**

Participants generally were well informed about each other's training and work activities and nearly everyone mentioned the importance of adequate communication and teamwork. Discrepancies were also observed. The other professions knew on average the least about the profession of anesthesiologists and most about the profession of surgeons. When assessing the responsibilities regarding tasks we found consensus in well-defined and/or protocolized tasks, but variation in less clearly defined tasks.

**Conclusions:**

Team- and task-related knowledge in the operating room team is reasonably well developed, but irregularly, with potentially crucial differences in knowledge related to patient care. Awareness of these discrepancies is the first step in further optimization of team performance.

## 1 Introduction

Health care is often delivered in frequently changing interprofessional teams. The operating room (OR) is a clear example: a highly complex environment with multiple involved professions, where good communication and collaboration are paramount. Issues impeding collaboration, such as communication errors, were frequently reported and can potentially lead to patient harm [1,2]. Discrepancies also exist between professions in perceptions of the quality of communication [3,4] and teamwork [4,5].

To communicate and function properly as a team, a shared mental model (SMM) is required [6,7]. Floren and colleagues proposed the following definition: *“A shared mental model is an individually held, organized, cognitive representation of task-related knowledge and/or team-related knowledge that is held in common among health care providers who must interact as a team in pursuit of common objectives for patient care.”* [8] It has been identified as an important element in team effectiveness [6,9]. Having a SMM positively influences the adaptability of a team, and reinforces behavior that supports other team members, such as mutual performance monitoring and back-up behavior [6].

Both the task-related and the team-related knowledge in a SMM [10] help shape behavior and expectations of team members. Task-related knowledge is knowledge about how a task is accomplished, and includes information about procedures, strategies, contingencies and environmental factors. Team-related knowledge can be related to individual team members (e.g., teammates' knowledge, skills, attitudes, preferences and tendencies) and to interactions within a team (e.g., roles and responsibilities, role interdependencies, information sources and flow, interaction patterns and communication channels) [10]. Mutual knowledge of work processes and professional roles is therefore key for effective collaborative practice [[11], [12], [13], [14]]. Lack of knowledge about other professions (i.e., lack of team-related knowledge) may lead to inefficient use of each other's capabilities [12,15]. Similarly, task-related knowledge, such as the responsibility for a specific task, has been shown to differ between professions [16]. This potentially impedes effective patient care, when tasks are performed multiple times, or not at all.

In demanding professional environments such as the OR, teams need to coordinate their actions effectively. Research has shown that SMMs contribute to team performance [17], because they create a framework that promotes common understanding and coordination, and in providing a mechanism for team leaders to regulate team performance [18]. The establishment of SMMs is facilitated when group members have accurate and shared information about group members’ knowledge and capabilities [19,20]. Increased attention for non-technical skills, which are closely related to SMMs, is reflected by the multitude of frameworks that are used in training health care professionals, such as the NOTSS [21], NOTECHS (II) [22] and TeamSTEPPS [23].

We aimed to explore potential differences in task- and team-related knowledge between disciplines. Members of the OR team from four professions (i.e. operation assistants, anesthesia assistants, surgical specialists and anesthesiologists) were asked about their knowledge regarding each other's training, work, and capabilities. Furthermore, we investigated what professionals consider traits of high-performing and underperforming colleagues of their own and of the other professions, and also how they allocate responsibilities in the OR in the Netherlands. Our hypothesis was that there are differences in task- and team-related knowledge between health care providers of different professions [16], which might impair the development of SMMs in the OR, which will negatively impact team performance.

## 2 Material and Methods

### 2.1 Study design

A single sample cross-sectional survey was conducted in three hospitals in the Netherlands, from September to October 2019. One of these was a tertiary academic center and the other two were regional teaching hospitals. The Medical Research Ethics Committee Utrecht deemed the study exempt from ethical review (proposal number 21/459). The research committee of the Division of Anesthesiology, Intensive Care and Emergency Medicine of the University Medical Center Utrecht required that all heads of departments were to be informed before the study was started and provided with the opportunity to request further information, which was done prior to sending the questionnaire. Information regarding the study was given at the beginning of the questionnaire. By participating in the questionnaire participants gave consent.

### 2.2 Study population

In the Netherlands, an operating room team consists of at least five persons of four professions: an anesthesiologist, a surgeon from a variety of specialties, two operation assistants (scrub nurses), and an anesthesia assistant. Anesthesia assistants work under supervision of an anesthesiologist, in a ‘two-table’ system in which the anesthesiologist supervises two ORs. Operation assistants either assist the surgeon with the surgery, or function as a circulating nurse. Operation assistants and anesthesia assistants are not necessarily nurses by background. Medical specialists and nurses are registered in the Dutch health care professions registry, meaning they are legally certified to work independently. Operation assistants and anesthesia assistants do not have this certification.

The research population for this study consisted of OR professionals and those in training to become OR professionals. Surgeons of the following specialties were invited to participate: general surgery, orthopedic surgery, gynecology, urology and otorhinolaryngology. These specialties were chosen because they are well represented in all general hospitals.

### 2.3 Data collection

A questionnaire (see Appendix 1) was developed by the researchers and subsequently tested by a group of representatives from each of the four professions. Based on recommendations by Burns and colleagues, each representative was asked to provide feedback on the content, clarity and relevance of the questionnaire items [24]. Adjustments were made based on their contributions. The first section of the questionnaire concerned demographics. To assess team-related knowledge, the questionnaire contained a section with questions about participants’ education and their daily activities and questions about potential traits of a high-performing and an underperforming colleague in the OR. To assess task-related knowledge the final section of the questionnaire was used to assess the opinion of the participants on the allocation of responsibilities in the OR, inspired by the method used by Steinemann and colleagues for trauma teams [25].

The questionnaire and information letter, asking for informed consent, were distributed online to every operation assistant, anesthesia assistant, surgical specialist and anesthesiologist, and trainees of each of these professions working in the participating hospitals. Formdesk, an online forms management system, was used to administer the questionnaire. A reminder was sent after four weeks. The distribution was done by the heads of departments and by the team leaders of the professionals involved. The questionnaire was voluntary. No incentives were offered and only completed questionnaires were analyzed.

### 2.4 Research instrument

The questionnaire consisted of three components.

#### 2.4.1 Team-related knowledge: knowledge about each other's work, activities and educational background

Participants were asked to indicate what they knew of the daily work, workplaces and training of all professions (including their own profession). For example: *What is the duration of anesthesia assistants' training?* We did not include questions regarding colleagues’ life outside of work. Although this might increase the quality of teamwork, since it differs between individuals, it is difficult to measure. Answers were scored as correct or incorrect for each item. The correct answers to the questions were identified based on training programs (for example, duration of training, compulsory internships) and by consulting with representatives of each profession. Multiple surgical specialties were invited to participate, so the questions regarding the surgical profession were designed to be applicable to all surgical specialties. For example, all surgical specialists receive five to six years of training. Answers regarding duration of training were therefore considered correct if they fell in this range. In order to be able to assess general knowledge professionals had about each profession, we elected to combine the questions about each profession into a total score. This was done by assigning a percentage to each answer, based on equal weight, adding up to 100 when all answers were correct. Individual items, such as the duration of the training, were considered less important, and thus we chose not to compare answers to individual questions between professions.

#### 2.4.2 Team-related knowledge: traits of the involved professions

Each participant was asked to name three traits of what *they* consider a high-performing colleague, and three traits of what can be considered as an underperforming colleague, for each of the four professions. The qualification of whether a trait was considered positive or negative was done by the participants.

#### 2.4.3 Task-related knowledge: responsibilities in the operating room

Participants were asked to assess 23 common tasks in and around the operating room, for example: ‘Preparing and checking the ventilator’ and ‘Performing the time-out procedure’. The tasks were selected by the researchers, after which they were judged by the representatives of each profession. The selected tasks were deemed a reflection of the perioperative process, although the list is not exhaustive. For each task the participants were asked to judge which profession was most responsible for executing that task. Although a lot of tasks are a joint responsibility, we elected to rank the relative responsibilities, as this reflects daily practice. Professions were sorted from 1 (most responsible) to 4 (least responsible). For the last five tasks ‘Organization outside the OR’ was included as a possibility to allocate responsibility.

### 2.5 Data analysis

Data analysis was done using IBM SPSS Statistics for Windows, Version 26.0.

The participant characteristics were analyzed descriptively.

#### 2.5.1 Team-related knowledge: knowledge about each other's work, activities and educational background

The mean percentage and standard deviation of correct answers per profession was calculated for each profession. Variance between groups was assessed using one-way ANOVA. The mean difference in knowledge scores was calculated by using the percentages of correct answers provided by professionals about their own profession as a reference score. Professions were compared using post hoc tests, specifically Games-Howell, since equal variances could not be assumed. Bootstrapping was performed because a normal distribution could not be assumed [26]. These results were expressed as mean difference and 95% confidence intervals.

#### 2.5.2 Team-related knowledge: traits of the involved professions

The mentioned traits were analyzed qualitatively and thematically grouped if they were similar in tendency (for example: “being precise” and “perfectionism”). The groups were descriptively analyzed and ranked according to the frequency they were mentioned. Three authors were involved in this analysis to ensure a uniform categorization of the qualities. Discrepancies or disagreements in the grouping of traits were discussed and resolved. The traits named by the professionals about their own profession were contrasted with those named by the three other professions.

#### 2.5.3 Task-related knowledge: responsibilities in the operating room

The Likert type scale was used to calculate means and 95% confidence intervals, in order to plot the distribution of perceived responsibilities. Means and medians were compared for each task and profession to ensure they were comparable in the ranking of the professions. This showed that means and medians were not too different.

We chose to use the mean to plot the distribution of responsibilities, because of the ease of interpretation. The distributions of the perceived responsibilities were plotted to help understanding.

## 3 Results

One hundred and six professionals responded. We estimate that the questionnaire was sent to approximately 500 professionals, giving a response rate of approximately 20%. The characteristics of the respondents are described in Table 1. All professions were represented, with professionals from surgical specialties being the minority (10%). Most respondents (77%) were certified professionals, the rest were in training for their respective professions. Almost half of the respondents have been working for over ten years in their respective professions.

**Table 1:** Characteristics of participants.

Profession	N (%)
Operation assistant	26 (25)
Of which in training	2 (2)
Anesthesia assistant	30 (28)
Of which in training	2 (2)
Surgical specialist	11 (10)
Of which in training	3 (3)
Anesthesiologist	39 (37)
Of which in training	17 (16)
**Years of practice within the profession (including time spent in training)**	
<5 years	36 (34)
5–10 years	22 (21)
>10 years	48 (45)
**Type of hospital**	
Academic center	74 (70)
Teaching hospital	32 (30)

Percentage of total number of participants (n = 106).

### 3.1 Team-related knowledge: knowledge about each other's work, activities and educational background

Table 2 provides the percentage of correct answers given by each profession regarding questions of team-related knowledge. All participants knew most about the level of training, educational program and daily work activities of their own profession. This can be seen by the percentages displayed on the diagonal of the upper half of Table 2. For example, on average operation assistants answered 92% of the questions about their own profession correctly. The percentages referring to knowledge about other professions are markedly lower, but still mostly around 80%. The surgical profession seems to be the best known, as all professions scored more than 83% regarding knowledge about surgeons, compared to scores below 80% and even below 70% for the other professions.

**Table 2:** Team–related knowledge regarding each profession (row), by each profession (column).

Mean knowledge score					
		Profession			
		OA	AA	Surg	Anesth
Knowledge regarding	OA	92 (9)	91 (9)	71 (15)	77 (15)
	AA	82 (13)	89 (7)	65 (20)	78 (12)
	Surg	83 (17)	88 (15)	94 (7)	91 (11)
	Anesth	66 (9)	75 (13)	76 (15)	91 (9)
**Differences between score and reference score**					
Knowledge regarding	OA	–	−1 (−6 to 3)	−21 (−30 to −12)	−15 (−20 to −9)
	AA	−7 (−13 to −1)	–	−24 (−36 to −12)	−11 (−16 to −7)
	Surg	−11 (18 to −4)	−6 (−12 to 0)	–	−4 (−8 to 2)
	Anesth	−25 (−29 to −21)	−16 (−21 to −11)	−15 (−24 to −7)	–

Based on Part 2 of the questionnaire (see Appendix 1). Upper half of the table: mean percentage (standard deviation) of correct answers. Lower half of the table: mean differences (95% confidence interval), between the reference score (score of own profession) and the score of each profession (upper half of table). OA = operation assistant, AA = anesthesia assistant, Surg = surgical specialist, Anesth = anesthesiologist.

Using one-way ANOVA we found a statistically significant difference between groups when considering knowledge regarding operation assistants, F (3,102) = 14.80, p < 0.001, anesthesia assistants, F (3,102) = 11.92, p < 0.001, and anesthesiologists, F (3,102) = 30.22, p < 0.001. The knowledge regarding surgeons did not statistically significantly differ between groups, F (3,102) = 2.34, p = 0.078.

Table 2 also shows the mean difference in knowledge scores compared to the reference score, i.e. the score of professionals about their own profession. For example, compared to the reference score, the knowledge of operation assistants about the profession of anesthesiologists is 25 points lower, with a 95% confidence interval of −29 to −21.

A majority of the mean differences in scores is statistically significant. Especially the professions of anesthesiologists and to a lesser extent anesthesia assistants were poorly known by their colleagues. As seen in Table 2, all three other professions scored significantly lower than the reference score regarding anesthesiologists. The same held true for the profession of anesthesia assistants. Table 2 shows similar scores for each profession regarding training and activities of surgeons, reflected by non-significant differences when compared to the reference scores.

### 3.2 Team-related knowledge: traits of the involved professions

Table 3 compares traits of high-performing and underperforming colleagues as stated by participants about their own profession with those mentioned by the other professions. The percentage of professionals of the disciplines involved that mention each quality is indicated. Adequate communication, good collaboration and teamwork were positive qualities mentioned by all participants regarding their own and each of the other professions.

**Table 3:** Traits of high- and underperforming colleagues for each profession.

Traits of high-performing colleagues				
	Own profession	%	Other three professions	%
OA	Adequate communication	56	Adequate communication	40
	Proactive and anticipating	36	Proactive and anticipating	31
	Knowledge	32	Stress-resistant	24
	Good collaboration and teamwork	24	Good collaboration and teamwork	23
AA	Adequate communication	45	Adequate communication	55
	Good collaboration and teamwork	24	Good collaboration and teamwork	30
	Proactive and anticipating	24	Stress-resistant	23
	Knowledge	24	Proactive and anticipating	20
Surg	Adequate communication	45	Adequate communication	68
	Stress-resistant	27	Good collaboration and teamwork	29
	Decisive	27	Skilled and competent	29
	Good collaboration and teamwork	27	Knowledge	17
			Stress-resistant	17
Anesth	Adequate communication	64	Adequate communication	53
	Good collaboration and teamwork	33	Stress-resistant	36
	Decisive	28	Good collaboration and teamwork	26
	Skilled and competent	18	Decisive	14
	Proactive and anticipating	18	Knowledge	14
	Stress-resistant	18		
Traits of underperforming colleagues				
OA	Disorganized and sloppy	44	Disorganized and sloppy	31
	Inadequate communication	40	Inadequate communication	28
	Insufficient knowledge	32	Individualistic	27
	Individualistic	28	Inflexible	13
AA	Inadequate communication	35	Disorganized and sloppy	35
	Disorganized and sloppy	21	Individualistic	34
	Individualistic	21	Inadequate communication	32
	Insufficient knowledge	14	Inflexible	12
Surg	Inadequate communication	45	Inadequate communication	52
	Individualistic	27	Individualistic	26
	Arrogant and authoritarian	27	Arrogant and authoritarian	18
	Insecure	18	Incompetent	17
	Inflexible	18		
	Aggressive and irritable	18		
Anesth	Inadequate communication	39	Inadequate communication	40
	Individualistic	33	Individualistic	20
	Disorganized and sloppy	28	Inflexible	12
	Panic in stressful situations	13	Incompetent	11

The four most often mentioned positive and negative qualities (or more in case of equal percentages) with percentage of participants mentioning these qualities. Of In the left column the opinion of participants about their own profession, in the right column the opinion of the other professions. OA = operation assistant, AA = anesthesia assistant, Surg = surgical specialist, Anesth = anesthesiologist.

Some traits were typically mentioned by participants concerning other professions, but not by the participants of the profession itself. Specifically, the following: stress-resistance (about operation and anesthesia assistants), being skilled and competent (about surgeons), and having adequate knowledge (about surgeons and anesthesiologists).

In contrast, several qualities were deemed important by participants about their own profession, but were not mentioned often by the other professionals, e.g. being well organized (operation assistants), having knowledge and being helpful (anesthesia assistants), being decisive (surgeons), being skilled and competent and being proactive and anticipating (anesthesiologists).

Traits of an underperforming colleague were more diverse, as indicated by the lower percentages in general. Inadequate communication and being individualistic were, similar to the positive qualities, mentioned about each profession, by all groups. Disorganized and sloppy were mentioned remarkably often, especially in relation to operation assistants and anesthesia assistants.

Other negative qualities often mentioned by the other professionals were: being inflexible (about operation assistants, anesthesia assistants and anesthesiologists) and being incompetent (about surgeons and anesthesiologists) Similar to the positive qualities, there were also characteristics that were little mentioned by colleagues, but have been mentioned often by respondents about their own profession. Examples are: surgeons mention being insecure, whereas the other professions do not mention this frequently in relation to surgeons. Similarly, anesthesiologists say that panicking in a stressful situation is perceived negatively, which is something that is not that often mentioned by the other professions.

### 3.3 Task-related knowledge: responsibilities in the OR

The participants in our study were asked to allocate responsibilities pertaining to 23 tasks. For several tasks the distribution of responsibility that emerges was similar for each profession, for example regarding the use of surgical instruments.

**Fig. 1:**
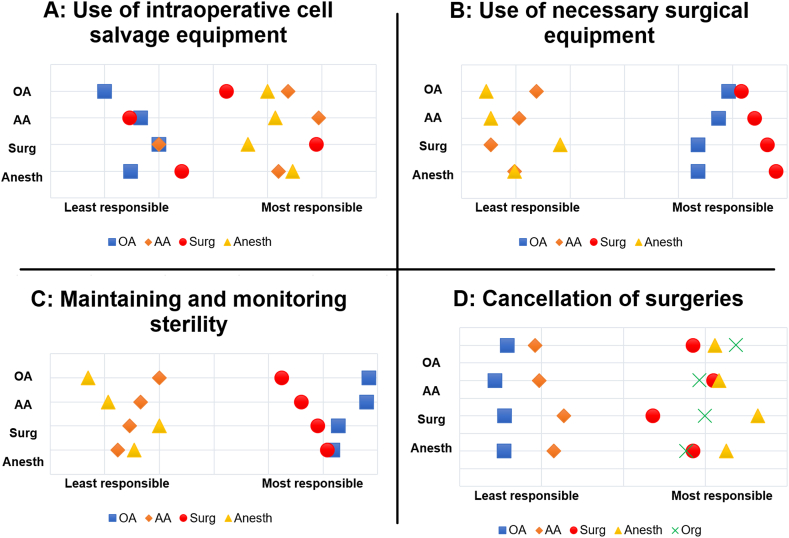
Allocation of responsibilities in specific tasks. Legend: Each profession (on the left) indicated how they considered the allocation of responsibilities for all professions. The following tasks are shown: the use of cell salvage equipment (panel A), the use of surgical equipment (panel B), maintaining and monitoring sterility (panel C), and cancellation of surgeries (panel D). OA = operation assistant, AA = anesthesia assistant, Surg = surgical specialist, Anesth = anesthesiologist, Org = organization outside the OR.

The allocation of responsibilities in other tasks was more diverse. Fig. 1 shows examples of general agreement (the use of surgical instruments, panel B, and maintaining and monitoring sterility, panel C), and also the most striking discrepancies, which occurred mainly in the use of the intraoperative cell salvage (panel A), positioning of the patient and cancellation of surgeries (panel D). For the distribution of all tasks see Appendix 2.

## 4 Discussion

We demonstrated differences in both team- and task-related knowledge. These could lead to friction and frustration between team members, which can be detrimental to patient care. Also, performance of tasks may be suboptimal, or tasks may not be performed at all [12,15]. Even though operating room personnel appeared generally well informed about each other's training and work activities and despite nearly all participants mentioning the importance of adequate communication and teamwork in their own and other professions.

For example, members of an anesthesia team were previously found to frequently be unaware of each other's capabilities, hampering effective allocation of tasks, leading to frustration and anxiety and putting the patient at risk [27]. Our findings indicate that all other professions relatively knew the least about the profession of anesthesiologists, and most about the profession of surgical specialists, as seen in Table 2. On average, the small sample of surgeons from different specialties in our study knew the least about the other professions, compared to the others. This might be partly attributable to the design of the questions, but is consistent with earlier findings, where surgeons overestimated their own knowledge regarding others' roles [28]. When looking at the separate professions it stands out that the scores were lower between operation assistants and anesthesiologists and between surgical specialists and anesthesia assistants. This could reflect a pattern of communication in the OR, where surgeons and operation assistants work more closely together, similar to anesthesiologists and anesthesia assistants. A previous study demonstrated this pattern by showing that not all interactions between each combination of professions were considered equally important by the participating OR professionals [28]. Our study indicates how multifaceted collaboration occurs in an OR-team. Further research into the interactions between the professions is required in order to determine where there is a potential risk to patient care, and where a focus could be for e.g. interprofessional simulation training.

Each profession noted the importance of communication and teamwork (see Table 3). There were, however, differences between the views of professionals on traits of high- and underperforming colleagues of their own profession compared to the view of their co-workers. An interesting example is that anesthesia assistants and operation assistants noted the importance of having “adequate knowledge”, whereas the other professions did not mention this. This might be something considered self-evident by anesthesiologists and surgeons, but seen as a defining quality for operation and anesthesia assistants. Negative traits that were mentioned, such as being incompetent, or being inflexible, do not immediately influence the team- or task-related knowledge, but are important in maintaining trust between team members, as well as influencing the adaptability of the team. These components are also defined as necessary for effective team performance, besides SMMs [6].

A subset of task-related knowledge was assessed by mapping perceived responsibilities for tasks in the operation room. Discrepancies in allocation of responsibilities can lead to inefficiency when activities are performed multiple times, or even dangerous situations for the patient when a necessary task is not performed at all. In our study, it appeared that clearly defined tasks, such as the ‘time-out’, were relatively straight-forward. There also was consensus regarding tasks concerning preparation and use of instruments and devices. However, for less protocolized responsibilities, e.g. positioning of the patient, the allotment was considerably less clear.

These results are in line with other studies, which also indicated differences in task-related knowledge in OR teams. For example, Nakarada-Kordic et al. assessed similarity in task-related knowledge in OR teams and found the lowest responsibility scores in optimal patient positioning and estimating blood loss. In half the tasks assessed there was poor agreement [16]. Different perceptions in roles and responsibilities were also found in a level II trauma center in the US, where trauma surgeons and nurses disagreed about the majority of resuscitation tasks [25]. In this context it should be noted that the meaning of ‘responsibility’ can be interpreted differently, for example, the difference between being responsible for the immediate execution of a task, and being ultimately responsible that a task is performed by someone, including the quality of the overall performance. In our study, we tried to distinguish between the profession that is responsible for task fulfillment and the profession or factor that exercises the most influence over the task. Furthermore, when allocating responsibilities in our study, participants might have interpreted tasks slightly different, for example, regarding ‘wellbeing of the patient during the surgery’. This was considered inherent to daily practice. Also, it is debatable whether all professions (need to) have the exact same level of task-related knowledge. Also, team-related knowledge is important in this regard. When task-related knowledge is not completely similar, aspects such as communication and allocation of roles and responsibilities are incredibly important, possibly compensating for a difference in task-related knowledge. The aim of optimizing SMMs is for each profession to know enough about the important aspects for the other professions to ensure smooth and effective collaboration. Wilson emphasizes the importance of communication and describes the practice of creating and enhancing shared mental models in daily practice, for example by starting the day with a team meeting, in which all interdisciplinary team members can introduce themselves, in which topics such as the time frame, required surgical instruments and patient positioning. Another tool to enhance the created SMM can be the WHO surgical patient safety checklist, in which for example the site of the procedure is confirmed [7].

Several limitations of this study must be acknowledged. The questionnaire used was checked by a group of representatives from the disciplines involved, from one participating center, providing face and content validity. The results show that for all professions, the participants know most about the training and work activities of their own profession. Because this is in line with what one would predict, this can serve as an indication for the validity of the knowledge questions. Psychometric analysis (e.g., factor or reliability analysis) was not performed, since the data were not suited to these techniques. Also, each individual question regarding knowledge of other professions might not be a proper indicator of knowledge on its own. We therefore chose to use a combined score in order to assess team-related knowledge. Regarding task-related knowledge, differentiating between either actual asymmetry in understanding of responsibilities or a slightly incidental allocation of responsibilities might prove difficult, especially in tasks that are a joint responsibility.

Completing the questionnaire was time-consuming, possibly deterring prospective participants from completing it. This may have led to a reduced response rate and also to selection bias, if only people who were keen on the subject participated. Furthermore, the representativeness of the sample might be limited. The sample of participating professionals was small, especially the surgical specialists. Besides being underrepresented in the researched population, they came from a variety of surgical disciplines. There was a relative overrepresentation of participants in the academic center, possibly because the research team was located in this center. This may limit generalizability. Also, task responsibility and allocation may differ between hospitals. We chose to include trainees in the questionnaire because they are, in daily practice, part of the team and thus influence team performance. Also, they might be even more aware of task- and team-related knowledge since they are learning about their own tasks and place within the OR team.

A lack of shared knowledge can induce risks in the OR. There are studies where increased similarity of the SMM positively influenced team performance [17,29]. However, too much similarity can lead to ‘groupthink’, which may discourage critical thinking [8]. It is as yet unclear which level of similarity is optimal for team performance.

Team- and task-related knowledge in the questioned OR teams was reasonably well developed, albeit irregular. Multiple differences were observed, with potential negative effects on patient care. Emergency situations arise relatively frequently in the OR, and knowing what e.g. an anesthesia assistant is capable of, can save minutes. Our findings may be used to increase awareness about differing SMMs among health care professionals and to optimize the development of SMMs. Further qualitative research could focus on the creation of SMMs in daily practice. In simulation the influence of specific factors in SMMs on team performance could be studied. The key is to find the optimal level of similarity in SMMs to ensure smooth collaboration and effective teamwork, which is especially important in a demanding work environment such as the OR.

## Funding

This research did not receive any specific grant from funding agencies in the public, commercial or not-for-profit sectors.

## Declaration of competing interest

The authors declare that they have no known competing financial interests or personal relationships that could have appeared to influence the work reported in this paper.
