# Supplementary material for: Team- and task-related knowledge in shared mental models in operating room teams: A survey study
Source: Heliyon. 2023 Jun 3;9(6):e16990. doi: 10.1016/j.heliyon.2023.e16990 (PMC10272475; doi:10.1016/j.heliyon.2023.e16990)
Supplement: Multimedia component 1 [file mmc1.docx]

SUPPLEMENTARY MATERIAL

APPENDIX A: Questionnaire (translated from original Dutch questionnaire)

**Part 1: Demographic questions**

1. What is your profession?
2. If you are a surgical specialist, in which specialty do you work?
3. Are you in training for your profession?
4. How long have you worked in your respective profession (including a possible training period)?
5. In which hospital do you work at the moment?

**Part 2: Knowledge about each other’s work, activities and educational background**

Regarding operation assistants:

1. What level of training do operation assistants receive?
2. What is the duration of their training?
3. Is the profession operation assistant recorded in the Dutch health care professions registry?
4. Are operation assistants nurses?
5. Indicate where operation assistants complete internships during their training (choose all that apply): cardiac and/or pulmonary function assessment clinic, radiology department, surgical ward, surgical outpatient clinic, intensive care unit, recovery ward, anesthesiology department, preoperative assessment clinic, pain management, sedation practitioners, central sterilisation department, infectious diseases management, OR planning
6. Indicate where operation assistants work (choose all that apply): central sterilisation department, operating room, recovery ward, surgical ward, surgical outpatient clinic, emergency department, intensive care unit

Regarding anesthesia assistants:

1. What level of training do anesthesia assistants receive?
2. What is the duration of their training?
3. Is the profession anesthesia assistant recorded in the Dutch health care professions registry?
4. Are anesthesia assistants nurses?
5. Indicate where anesthesia assistants complete internships during their training (choose all that apply): laboratory and/or blood bank, cardiac and/or pulmonary function assessment clinic, radiology department, surgical ward, surgical outpatient clinic, intensive care unit, recovery ward, preoperative assessment clinic, pain management, sedation practitioners, central sterilisation department, infectious diseases management, OR planning
6. Indicate where anesthesia assistants work (choose all that apply): operating room, recovery ward, surgical ward, surgical outpatient clinic, emergency department, intensive care unit, preoperative assessment clinic, pain management, medical ward, cardiac and/or pulmonary function assessment clinic

Regarding surgical specialists:

1. What level of training do surgical specalists receive?
2. What is the duration of their training?
3. Is the specialty recorded in the Dutch health care professions registry?
4. Indicate where surgical specialists work (choose all that apply): operating room, recovery ward, surgical ward, surgical outpatient clinic, emergency department, intensive care unit, cardiac and/or pulmonary function assessment clinic, central sterilisation department

Regarding anesthesiologists:

1. What level of training do anesthesiologists receive?
2. What is the duration of their training?
3. Is the specialty recorded in the Dutch health care professions registry?
4. Indicate where anesthesiologists complete internships during their training (choose all that apply): pediatric anesthesiology, pain management, intensive care unit, recovery ward, cardiology department, pulmonary medicine, preoperative assessment clinic, surgical department, academic hospital, regional hospital
5. Indicate where anesthesiologists work (choose all that apply): operating room, recovery ward, surgical and/or medical ward, emergency department, intensive care unit, preoperative assessment clinic, pain management outpatient clinic, cardiac and/or pulmonary function assessment clinic, central sterilisation department

**Part 3: Traits of the involved professions**

Name three traits or qualities of a well-functioning colleague in the OR, for your own profession and the other three professions. Try to be as specific as possible.

Name three traits or qualities of a malfunctioning colleague in the OR, for your own profession and the other three professions. Try to be as specific as possible.

**Part 4: Responsibilities in the OR**

Responsibilities can be interpreted in different ways. For the following questions responsibility is defined as the profession which is responsible for immediate task fulfillment and the quality of the performance. For each task, indicate which profession is the most responsible (1), the most responsible after that (2), a little responsible (3) and the least responsible (4). Use each number once.

1. Checking the air pressure in the OR
2. Preparing and checking laparoscopic video equipment
3. Using laparoscopic video equipment
4. Preparing and checking the ventilator
5. Using the ventilator
6. Preparing and checking intraoperative cell salvage equipment
7. Use of intraoperative cell salvage equipment
8. Preparing and checking surgical instruments
9. Use of necessary surgical instruments
10. Performing the time-out procedure
11. Communication with the patient
12. Wellbeing of the patient, before and after anesthesia
13. Positioning the patient
14. Maintaining and monitoring sterility
15. Wellbeing of the patient during the surgery
16. Counting the materials used
17. Performing the sign to out procedure
18. Postoperative handover to the recovery
19. General quality of care in the operating room

For the following tasks responsibility means the profession or factor that exercises the most influence over the task.

1. Duration of surgery
2. Use of available operating rooms
3. Exceeding planned OR time
4. Cancellation of surgery
5. Planning emergency surgery

APPENDIX B: Allocation of responsibilities

For each task each profession allocated responsibility to their own and the other professions. Reported are means and 95% confidence intervals per profession by each profession, with 1 being the most responsible and 4 being the least responsible for the specific task. For task number 19-23 responsibility is defined as ‘whoever exercises the most influence over the mentioned task’, and includes the organization/logistics outside of the operating room.

Abbreviations: OA = operation assistant, AA = anesthesia assistant, Surg = surgical specialist, Anesth = anesthesiologist.

|  | **Responsibility of profession:** | | | |
| --- | --- | --- | --- | --- |
|  | **OA** | **AA** | **Surg** | **Anesth** |
| Task 1: Checking the air pressure in the OR | | |  |  |
| OA | 1.54 (1.19 to 1.89) | 2.85 (2.51 to 3.18) | 2.12 (1.71 to 2.52) | 3.31 (2.97 to 3.65) |
| AA | 1.63 (1.35 to 1.92) | 3.00 (2.61 to 3.39) | 2.03 (1.62 to 2.44) | 3.10 (2.76 to 3.44) |
| Surg | 1.91 (1.15 to 2.67) | 2.91 (2.15 to 3.67) | 2.18 (1.40 to 2.97) | 2.73 (2.20 to 3.26) |
| Anesth | 1.82 (1.50 to 2.14) | 3.41 (3.13 to 3.69) | 1.56 (1.31 to 1.82) | 2.90 (2.64 to 3.15) |
| Task 2: Preparing and checking laparoscopic video equipment | | | |  |
| OA | 1.00 (1.00 to 1.00) | 3.12 (2.88 to 3.35) | 2.19 (2.03 to 2.35) | 3.92 (3.81 to 4.03) |
| AA | 1.03 (0.97 to 1.10) | 3.23 (3.02 to 3.45) | 2.07 (1.93 to 2.20) | 3.87 (3.74 to 4.00) |
| Surg | 1.00 (1.00 to 1.00) | 3.45 (2.99 to 3.92) | 2.09 (1.89 to 2.29) | 3.36 (2.91 to 3.82) |
| Anesth | 1.08 (0.99 to 1.16) | 3.26 (3.08 to 3.43) | 1.90 (1.75 to 2.04) | 3.72 (3.57 to 3.87) |
| Task 3: Using laparoscopic video equipment | | | |  |
| OA | 1.54 (1.33 to 1.74) | 3.31 (3.12 to 3.50) | 1.46 (1.26 to 1.67) | 3.85 (3.70 to 3.99) |
| AA | 1.60 (1.41 to 1.79) | 3.23 (2.94 to 3.52) | 1.53 (1.26 to 1.81) | 3.80 (3.65 to 3.95) |
| Surg | 1.73 (1.29 to 2.16) | 3.64 (3.18 to 4.09) | 1.27 (0.96 to 1.59) | 3.18 (2.78 to 3.59) |
| Anesth | 1.59 (1.43 to 1.75) | 3.38 (3.22 to 3.54) | 1.33 (1.18 to 1.49) | 3.67 (3.51 to 3.82) |
| Task 4: Preparing and checking the ventilator | | |  |  |
| OA | 3.62 (3.41 to 3.82) | 1.08 (0.97 to 1.19) | 3.46 (3.23 to 3.70) | 1.96 (1.72 to 2.20) |
| AA | 3.50 (3.25 to 3.75) | 1.23 (1.00 to 1.47) | 3.57 (3.35 to 3.78) | 1.83 (1.61 to 2.05) |
| Surg | 3.82 (3.41 to 4.22) | 1.36 (1.02 to 1.70) | 3.00 (2.70 to 3.30) | 1.64 (1.18 to 2.09) |
| Anesth | 3.54 (3.37 to 3.70) | 1.13 (0.96 to 1.30) | 3.41 (3.20 to 3.62) | 1.87 (1.76 to 1.98) |
| Task 5: Using the ventilator | | |  |  |
| OA | 3.73 (3.55 to 3.91) | 1.62 (1.41 to 1.82) | 3.19 (2.94 to 3.45) | 1.38 (1.10 to 1.67) |
| AA | 3.47 (3.21 to 3.72) | 1.63 (1.38 to 1.88) | 3.50 (3.29 to 3.71) | 1.27 (1.10 to 1.43) |
| Surg | 3.55 (2.85 to 4.24) | 1.91 (1.35 to 2.47) | 3.00 (2.70 to 3.30) | 1.27 (0.96 to 1.59) |
| Anesth | 3.74 (3.60 to 3.89) | 1.92 (1.81 to 2.04) | 3.28 (3.13 to 3.43) | 1.05 (0.98 to 1.12) |
| Task 6: Preparing and checking intraoperative cell salvage equipment | | | |  |
| OA | 3.27 (2.98 to 3.56) | 1.12 (0.94 to 1.29) | 3.31 (3.03 to 3.58) | 2.08 (1.80 to 2.35) |
| AA | 3.20 (2.88 to 3.52) | 1.23 (0.98 to 1.49) | 3.63 (3.45 to 3.82) | 1.93 (1.72 to 2.15) |
| Surg | 2.73 (1.82 to 3.63) | 2.00 (1.33 to 2.67) | 2.73 (2.12 to 3.33) | 2.27 (1.53 to 3.01) |
| Anesth | 3.23 (3.03 to 3.43) | 1.08 (0.99 to 1.16) | 3.46 (3.27 to 3.66) | 1.97 (1.82 to 2.13) |
| Task 7: Use of intraoperative cell salvage equipment | | | |  |
| OA | 3.50 (3.19 to 3.81) | 1.81 (1.46 to 2.15) | 2.38 (1.91 to 2.86) | 2.00 (1.64 to 2.36) |
| AA | 3.17 (2.83 to 3.51) | 1.53 (1.20 to 1.87) | 3.27 (1.91 to 2.86) | 1.93 (1.64 to 2.23) |
| Surg | 3.00 (2.26 to 3.74) | 3.00 (2.33 to 3.67) | 1.55 (0.99 to 2.10) | 2.18 (1.59 to 2.77) |
| Anesth | 3.26 (3.04 to 3.48) | 1.90 (1.55 to 2.24) | 2.79 (2.43 to 3.16) | 1.77 (1.54 to 2.00) |
| Task 8: Preparing and checking surgical instruments | | | |  |
| OA | 1.00 (1.00 to 1.00) | 3.19 (2.96 to 3.42) | 2.19 (1.99 to 2.39) | 3.85 (3.70 to 3.99) |
| AA | 1.00 (1.00 to 1.00) | 3.47 (3.28 to 3.66) | 1.93 (1.84 to 2.03) | 3.77 (3.61 to 3.93) |
| Surg | 1.18 (0.78 to 1.59) | 3.45 (2.99 to 3.92) | 1.91 (1.71 to 2.11) | 3.27 (2.74 to 3.80) |
| Anesth | 1.05 (0.98 to 1.12) | 3.51 (3.33 to 3.69) | 1.95 (1.82 to 2.08) | 3.51 (3.35 to 3.68) |
| Task 9: Use of necessary surgical instruments | | |  |  |
| OA | 1.54 (1.33 to 1.74) | 3.31 (3.12 to 3.50) | 1.42 (1.22 to 1.63) | 3.77 (3.60 to 3.94) |
| AA | 1.63 (1.43 to 1.84) | 3.47 (3.28 to 3.66) | 1.30 (1.13 to 1.47) | 3.73 (3.57 to 3.90) |
| Surg | 1.82 (1.55 to 2.09) | 3.73 (3.29 to 4.16) | 1.18 (0.91 to 1.45) | 3.09 (2.73 to 3.45) |
| Anesth | 1.82 (1.69 to 1.95) | 3.51 (3.33 to 3.69) | 1.10 (0.98 to 1.23) | 3.51 (3.35 to 3.68) |
| Task 10: Performing the time to out procedure | | | |  |
| OA | 2.65 (2.21 to 3.10) | 3.00 (2.53 to 3.47) | 1.12 (0.98 to 1.25) | 1.81 (1.55 to 2.06) |
| AA | 3.00 (2.63 to 3.37) | 3.00 (2.65 to 3.35) | 1.00 (1.00 to 1.00) | 1.87 (1.55 to 2.06) |
| Surg | 2.73 (1.93 to 3.53) | 3.00 (2.21 to 3.79) | 1.27 (0.84 to 1.71) | 1.91 (1.35 to 2.47) |
| Anesth | 3.23 (2.98 to 3.48) | 3.38 (3.12 to 3.65) | 1.08 (0.99 to 1.16) | 1.79 (1.66 to 1.93) |
| Task 11: Wellbeing of the patient, before and after anesthesia | | | |  |
| OA | 3.62 (3.25 to 3.98) | 2.04 (1.71 to 2.37) | 2.27 (1.94 to 2.60) | 1.35 (1.12 to 1.57) |
| AA | 3.57 (3.31 to 3.82) | 1.87 (1.61 to 2.12) | 2.70 (2.40 to 3.00) | 1.17 (1.03 to 1.31) |
| Surg | 3.73 (3.29 to 4.16) | 1.82 (1.41 to 2.22) | 2.55 (1.99 to 3.10) | 1.18 (0.91 to 1.45) |
| Anesth | 3.85 (3.71 to 3.99) | 2.26 (2.09 to 2.42) | 2.49 (2.24 to 2.73) | 1.08 (0.99 to 1.16) |
| Task 12: Positioning the patient | | | |  |
| OA | 2.88 (2.44 to 3.33) | 2.12 (1.75 to 2.48) | 1.27 (1.05 to 1.48) | 2.46 (1.99 to 2.94) |
| AA | 3.13 (2.70 to 3.57) | 2.23 (1.88 to 2.58) | 1.37 (1.08 to 1.65) | 1.83 (1.49 to 2.17) |
| Surg | 3.00 (2.33 to 3.67) | 3.00 (2.48 to 3.52) | 1.18 (0.91 to 1.45) | 2.55 (1.85 to 3.24) |
| Anesth | 3.72 (3.50 to 3.94) | 2.67 (2.43 to 2.91) | 1.56 (1.32 to 1.81) | 1.54 (1.34 to 1.73) |
| Task 13: Maintaining and monitoring sterility | | | |  |
| OA | 1.08 (0.97 to 1.19) | 3.00 (2.84 to 3.16) | 1.88 (1.71 to 2.06) | 3.65 (3.35 to 3.95) |
| AA | 1.10 (0.99 to 1.21) | 3.17 (2.89 to 3.45) | 1.70 (1.53 to 1.87) | 3.47 (3.21 to 3.72) |
| Surg | 1.36 (1.02 to 1.70) | 3.27 (2.59 to 3.95) | 1.55 (1.19 to 1.90) | 3.00 (2.40 to 3.60) |
| Anesth | 1.41 (1.25 to 1.57) | 3.38 (3.14 to 3.63) | 1.46 (1.30 to 1.63) | 3.23 (2.99 to 3.47) |
| Task 14: Wellbeing of the patient during the surgery | | |  |  |
| OA | 3.35 (2.92 to 3.77) | 1.81 (1.45 to 2.17) | 2.31 (1.99 to 2.63) | 1.31 (1.09 to 1.53) |
| AA | 3.47 (3.13 to 3.80) | 1.47 (1.25 to 1.68) | 2.87 (2.58 to 3.16) | 1.40 (1.19 to 1.61) |
| Surg | 3.55 (2.85 to 4.24) | 2.09 (1.53 to 2.65) | 2.09 (1.46 to 2.73) | 1.36 (1.02 to 1.70) |
| Anesth | 3.64 (3.36 to 3.92) | 2.13 (1.92 to 2.34) | 2.49 (2.22 to 2.75) | 1.05 (0.98 to 1.12) |
| Task 15: Counting the materials used | | | |  |
| OA | 1.00 (1.00 to 1.00) | 3.35 (3.15 to 3.54) | 2.08 (1.92 to 2.24) | 3.73 (3.52 to 3.95) |
| AA | 1.10 (0.99 to 1.21) | 3.40 (3.19 to 3.61) | 1.73 (1.54 to 1.93) | 3.63 (3.45 to 3.82) |
| Surg | 1.00 (1.00 to 1.00) | 3.73 (3.29 to 4.16) | 2.00 (2.00 to 2.00) | 3.09 (2.73 to 3.45) |
| Anesth | 1.23 (1.04 to 1.42) | 3.44 (3.26 to 3.61) | 1.74 (1.58 to 1.91) | 3.38 (3.17 to 3.60) |
| Task 16: Performing the sign to out procedure | | | |  |
| OA | 2.42 (1.99 to 2.85) | 2.81 (2.40 to 3.22) | 1.04 (0.96 to 1.12) | 2.46 (2.03 to 2.89) |
| AA | 2.90 (2.52 to 3.28) | 2.83 (2.51 to 3.16) | 1.07 (0.97 to 1.16) | 2.23 (1.90 to 2.57) |
| Surg | 2.64 (1.88 to 3.39) | 3.09 (2.39 to 3.79) | 1.09 (0.89 to 1.29) | 2.36 (1.82 to 2.91) |
| Anesth | 3.13 (2.86 to 3.40) | 3.28 (3.00 to 3.56) | 1.05 (0.98 to 1.12) | 1.97 (1.80 to 2.15) |
| Task 17: Postoperative handover to the recovery | | | |  |
| OA | 3.92 (3.81 to 4.03) | 1.65 (1.35 to 1.95) | 2.77 (2.56 to 2.98) | 1.46 (1.23 to 1.70) |
| AA | 3.80 (3.57 to 4.03) | 1.93 (1.66 to 2.21) | 2.90 (2.67 to 3.13) | 1.13 (1.00 to 1.26) |
| Surg | 3.64 (3.09 to 4.18) | 2.00 (1.40 to 2.60) | 2.73 (2.41 to 3.04) | 1.36 (0.91 to 1.82) |
| Anesth | 3.92 (3.84 to 4.01) | 2.18 (2.02 to 2.34) | 2.77 (2.48 to 2.90) | 1.03 (0.97 to 1.08) |
| Task 18: General quality of care in the operating room | | | |  |
| OA | 2.62 (2.07 to 3.16) | 2.50 (2.05 to 2.95) | 1.50 (1.15 to 1.85) | 1.77 (1.39 to 2.15) |
| AA | 2.70 (2.21 to 3.19) | 2.27 (1.86 to 2.67) | 1.57 (1.23 to 1.90) | 1.73 (1.39 to 2.07) |
| Surg | 2.91 (2.15 to 3.67) | 3.09 (2.46 to 3.73) | 1.18 (0.91 to 1.45) | 1.91 (1.35 to 2.47) |
| Anesth | 3.38 (3.07 to 3.70) | 2.95 (2.68 to 3.22) | 1.41 (1.25 to 1.57) | 1.44 (1.27 to 1.60) |

|  | **OA** | **AA** | **Surg** | **Anesth** | **Organization** |
| --- | --- | --- | --- | --- | --- |
| Task 19: Duration of surgery | | | |  |  |
| OA | 3.23 (2.86 to 3.60) | 3.73 (3.36 to 4.10) | 1.04 (0.96 to 1.12) | 2.50 (2.13 to 2.87) | 3.73 (3.11 to 4.35) |
| AA | 3.07 (2.64 to 3.49) | 3.47 (3.10 to 3.83) | 1.00 (1.00 to 1.00) | 2.87 (2.47 to 3.27) | 3.83 (3.31 to 4.35) |
| Surg | 3.09 (2.39 to 3.79) | 3.45 (2.83 to 4.08) | 1.09 (0.89 to 1.29) | 2.64 (2.09 to 3.18) | 4.18 (3.19 to 5.17) |
| Anesth | 3.13 (2.75 to 3.51) | 3.79 (3.48 to 4.11) | 1.00 (1.00 to 1.00) | 2.49 (2.21 to 2.76) | 3.77 (3.32 to 4.22) |
| Task 20: Use of available operating rooms | | | |  |  |
| OA | 4.08 (3.62 to 4.53) | 3.92 (3.60 to 4.24) | 2.62 (2.25 to 2.98) | 2.69 (2.42 to 2.97) | 1.00 (1.00 to 1.00) |
| AA | 4.40 (4.07 to 4.73) | 4.23 (3.98 to 4.49) | 2.53 (2.18 to 2.88) | 2.40 (2.17 to 2.63) | 1.07 (0.93 to 1.20) |
| Surg | 3.91 (3.04 to 4.78) | 4.00 (3.33 to 4.67) | 3.00 (2.40 to 3.60) | 2.36 (1.61 to 3.12) | 1.18 (0.78 to 1.59) |
| Anesth | 4.26 (3.95 to 4.56) | 3.87 (3.56 to 4.18) | 2.59 (2.29 to 2.88) | 2.18 (1.92 to 2.44) | 1.54 (1.15 to 1.93) |
| Task 21: Exceeding planned OR time | | | |  |  |
| OA | 3.77 (3.35 to 4.19) | 3.85 (3.44 to 4.25) | 1.00 (1.00 to 1.00) | 2.38 (2.00 to 2.76) | 3.15 (2.55 to 3.76) |
| AA | 4.13 (3.78 to 4.48) | 3.77 (3.48 to 4.06) | 1.00 (1.00 to 1.00) | 2.77 (2.42 to 3.12) | 2.93 (2.42 to 3.45) |
| Surg | 3.27 (2.53 to 4.01) | 3.64 (2.95 to 4.33) | 1.09 (0.89 to 1.29) | 2.27 (1.74 to 2.80) | 3.64 (2.50 to 4.77) |
| Anesth | 3.56 (3.22 to 3.91) | 3.85 (3.55 to 4.14) | 1.05 (0.98 to 1.12) | 2.46 (2.23 to 2.69) | 3.46 (2.99 to 3.93) |
| Task 22: Cancellation of surgery | | | |  |  |
| OA | 4.38 (4.00 to 4.76) | 4.15 (3.76 to 4.54) | 1.92 (1.58 to 2.26) | 2.12 (1.79 to 2.45) | 1.73 (1.24 to 2.22) |
| AA | 4.70 (4.50 to 4.90) | 4.20 (3.95 to 4.45) | 1.57 (1.33 to 1.80) | 2.30 (2.00 to 2.60) | 1.90 (1.52 to 2.28) |
| Surg | 4.09 (3.27 to 4.91) | 3.55 (2.85 to 4.24) | 3.27 (2.47 to 4.07) | 2.27 (1.47 to 3.07) | 1.09 (0.89 to 1.29) |
| Anesth | 4.54 (4.24 to 4.83) | 4.03 (3.77 to 4.28) | 1.95 (1.62 to 2.28) | 2.41 (1.62 to 2.28) | 1.82 (1.46 to 2.18) |
| Task 23: Planning emergency surgery | | | |  |  |
| OA | 4.42 (3.99 to 4.85) | 4.08 (3.70 to 4.45) | 2.15 (1.84 to 2.47) | 1.88 (1.60 to 2.17) | 1.62 (1.19 to 2.04) |
| AA | 4.57 (4.26 to 4.87) | 4.03 (3.75 to 4.32) | 1.90 (1.60 to 2.20) | 1.83 (1.55 to 2.11) | 2.07 (1.61 to 2.53) |
| Surg | 4.45 (3.76 to 5.15) | 3.73 (3.05 to 4.41) | 2.64 (1.83 to 3.45) | 1.36 (1.02 to 1.70) | 2.00 (1.26 to 2.74) |
| Anesth | 4.46 (4.19 to 4.73) | 3.85 (3.59 to 4.10) | 2.15 (1.88 to 2.43) | 1.74 (1.50 to 1.99) | 2.23 (1.79 to 2.67) |
